# Dissipation and Residues of Imidacloprid and Its Efficacy against Whitefly, *Bemisia tabaci*, in Tomato Plants under Field Conditions

**DOI:** 10.3390/molecules27217607

**Published:** 2022-11-06

**Authors:** Manal A. A. Abdel razik, Zamzam M. Al Dhafar, Aisha M. Alqahtani, Mohamed A. Osman, Mohamed E. Sweelam

**Affiliations:** 1 Pesticides Department, Faculty of Agriculture, Menoufia University, Shebin El-Kom P.O. Box 32514, Egypt; 2 Department of Biology, College of Science, Imam Abdulrahman Bin Faisal University, P.O. Box 1982, Dammam 31441, Saudi Arabia; 3 Basic and Applied Scientific Research Center (BASRC), Imam Abdulrahman Bin Faisal University, P.O. Box 1982, Dammam 31441, Saudi Arabia; 4 Economic Entomology & Agricultural Zoology Department, Faculty of Agriculture, Menoufia University, Shebin El-Kom P.O. Box 32514, Egypt

**Keywords:** neonicotinoids insecticide, sucking insects, vegetables, analysis, *Solanum lycopersicum*

## Abstract

The whitefly, *Bemisia tabaci*, is the main pest for many field and horticultural crops, causing main and significant problems. The efficiency of imidacloprid insecticide as seed treatment and foliar spray at three rates against the whitefly, *B. tabaci*, was evaluated in tomato plants under field conditions; in addition, insecticide residues were determined in tomato leaves and fruits. The obtained results revealed that the seedlings produced from treated seeds with imidacloprid were the most effective treatment in decreasing whitefly stages. Reduction percentages of whitefly stages in seedlings produced from treated seeds and sprayed with ½, ¾ and 1 field rates of imidacloprid were more than that produced from untreated seeds. Tomato fruit yield in seedlings produced from treated seeds and sprayed with one recommended rate of imidacloprid was more than that of untreated seeds. The residues of imidacloprid in leaves and fruits in seedlings produced from treated seeds and sprayed with field rate were more than that of untreated seeds; additionally, the residues were higher in leaves than in fruits. The residual level in fruits was less than the maximum residual level (MRL = 1 mg kg^−1^) of the Codex Alimentarius Commission. The half-life (t ½) was 6.99 and 6.48 days for leaves and fruits of seedlings produced from treated seeds and 5.59 and 4.59 days for untreated seeds. Residues in tomato fruits were less than the MRL, therefore, imidacloprid is considered an unconventional insecticide appropriate for *B. tabaci* control that could be safe for the environment.

## 1. Introduction

The tomato, *Solanum lycopersicum* L., is considered to be one of the most prevalent and common vegetables in the world [1]. Tomato plants are infested with many pests and diseases, especially piercing sucking insects such as the aphid and whitefly, which play a vital role in transmitting plant diseases. The whitefly, *Bemisia tabaci*, is the main severe pest for many field crops [2,3]. Adult and nymphal stages of whitefly attack plants by sucking sap cells and secreting honeydew that inhibits the photosynthesis process and leads to reduced quality and quantity of yield [4,5]. The effective control of *B. tabaci* on vegetables depends mainly on chemical insecticides, particularly neonicotinoids [6]. Imidacloprid is a nicotine-based systemic neurotoxin insecticide [7]. Imidacloprid was the first neonicotinoid seed treatment insecticide used to protect seeds and seedlings against injury by early season insects [8,9,10,11,12].All imidacloprid treatments resulted in a substantial decrease in adult and immature whiteflies on the plants [13]. Imidacloprid protected cotton plants from thrips and jassid for up to 8 weeks [14]. Imidacloprid is mainly applied as seed dressing formulation [15]. The field residue trials with imidacloprid after seed-dressing of sunflower, corn and rape revealed no residues above the standard limits in pollen and nectar. Imidacloprid was effective against thrips for 7 weeks after planting [16]. Seedling root treatment before planting with imidacloprid achieved more protection from whitefly stage infection than seed treatment, and protected plants from infection for 7–8 weeks in the case of seed treatment [17]. spraying thiamethoxam and imidacloprid insecticides reduced the population of sucking insect pests in field crops [18]. In 1998, imidacloprid was used successfully to control *Bemisia argentifolii* on tomato in South Florida [19]. The residues of imidacloprid were dissipated below the maximum residue limit (MRL) of 1 mg kg^−1^ in 3 days, and reported that the half-life (t ½) for imidacloprid in cucumber was 3.40 and 2.70 days at single and double doses, respectively [20]. Imidacloprid has a minimum preharvest interval (PHI) of 3.8 days and was the most toxic against sweet potato whitefly adults, at 98.7% [21]. In addition, [22] found that imidacloprid and acephate caused the lowest recorded *B. tabaci* population (1.88 and 2.41; 1.99 and 2.53/3leaf/plant) during the first and second spraying. Recently, [23] found that the *B. tabaci* population was greatly reduced after 7 days of spraying cucumber with imidacloprid insecticide.

From the previous studies and our article [17], where tomato seeds, variety Beto 86, were used to compare between seedling, root and seed treatment on immature stages of whitefly, *B. tabaci*, with the additional aim of discovering the effect of foliar spray at half and one recommended rates of imidacloprid on the egg, nymph and adult stage, the obtained results show that the two treatment methods were effective on the whitefly and that STRs were more effective than seed treatment. Moreover, the residues of imidacloprid in the leaves and fruits of tomato STRs and sprayed with the recommended rate of imidacloprid compared with SURs sprayed with recommended rate of imidacloprid were determined, and it was reported that the residues in leaves and fruits produced from STRs and sprayed with imidacloprid were more than in those of untreated leaves and fruits sprayed with imidacloprid. Additionally, less residue was found in fruits than in leaves. Seed treatment is considered to be an important, safe and effective method of treatment; therefore, our previous study [17] was conducted based on the treated seeds method. The present work plans to cast light on the effect of tomato seed treatment on whitefly stages at different periods of treatment, as well as compare between the effect of tomato STSs (tomato seeds, variety Super Strain B, which produce good yields and are suitable for the experiment period) sprayed with 1/2, 3/4 and 1 recommended rates of imidacloprid and SUSs sprayed with three rates of imidacloprid on the stages of the whitefly, *B. tabaci*, to determine the best method of treatment, achieve effective whitefly control, minimize spraying numbers, and reduce environmental pollution and human toxicity. Determination of the residues of imidacloprid in the fruits and leaves of tomato STSs and SUSs and sprayed with the field recommended rate was conducted in order to determine the residues after different periods from spraying, as well as the PHI period and MRL.

## 2. Results and Discussion

**Figure 1:**
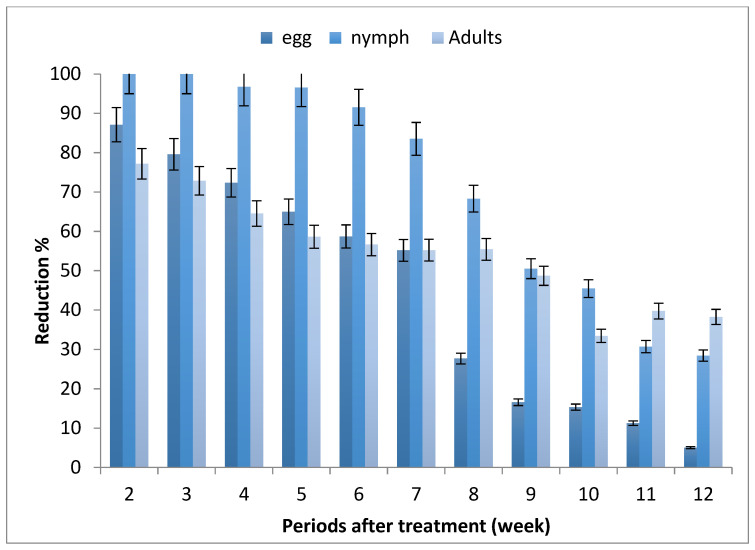
Effect of tomato seed treatment with imidacloprid on whitefly, *Bemisia tabaci*, stages.

The effect of tomato seed treatment with imidacloprid on the population density of whitefly, *Bemisia tabaci*, stages in the field:

The results depicted in Figure 1 revealed that the mean numbers of whitefly stages increased along the experimental period, where the highest number of eggs, nymphs and adults were recorded after 7, 10 and 9 weeks from planting, respectively.

The reduction percentages of *B. tabaci* infested tomato leaves of seedlings produced from the treated seeds were decreased by increasing the periods after planting, and ranged between (5.05–87.10%), (28.44–100%) and (33.42–77.18%) for egg, nymph and adult stages, respectively.

Generally, the obtained data show that seedling produced from treated seeds (STSs) led to the protection of tomato plants from whitefly infestation for 7–10 weeks after sowing, especially nymph stages.

The results of this study adequately agree with those of [24], who reported that imidacloprid as a seed treatment protected cotton seedlings from whitefly for up to 10 weeks, while [16,18] found that imidacloprid was effective against whiteflies for 6–7 weeks after cultivation. It was reported by [8,9,10,11,12] that imidacloprid protected seeds and seedlings against early-season insects when used as a seed treatment. Ref. [25] found that imidacloprid and thiamethoxam seed treatment decreased the population of *B. tabaci* below the economic threshold level up to 50 days after sowing compared with controls. In addition, [26] found that seed treatment of imidacloprid induced a fast initial effect on immature stages of whitefly, which gradually decreased to reach a moderate effect; in addition, a moderate initial reduction was observed on mature stages of whitefly. Furthermore, the obtained results were confirmed by [27], who found that imidacloprid exhibited relatively fast initial effects with long residual action against immature stages of whitefly and moderate effects on adults.

The effect of three rates of imidacloprid spraying on tomato seedlings produced from treated seeds against *Bemisia tabaci* in the field:

In this experiment, the effect of spraying imidacloprid insecticide at three rates (1/2, 3/4 and one rates) on tomato STSs against whitefly stages (egg, nymph and adults) were evaluated.

**Figure 2:**
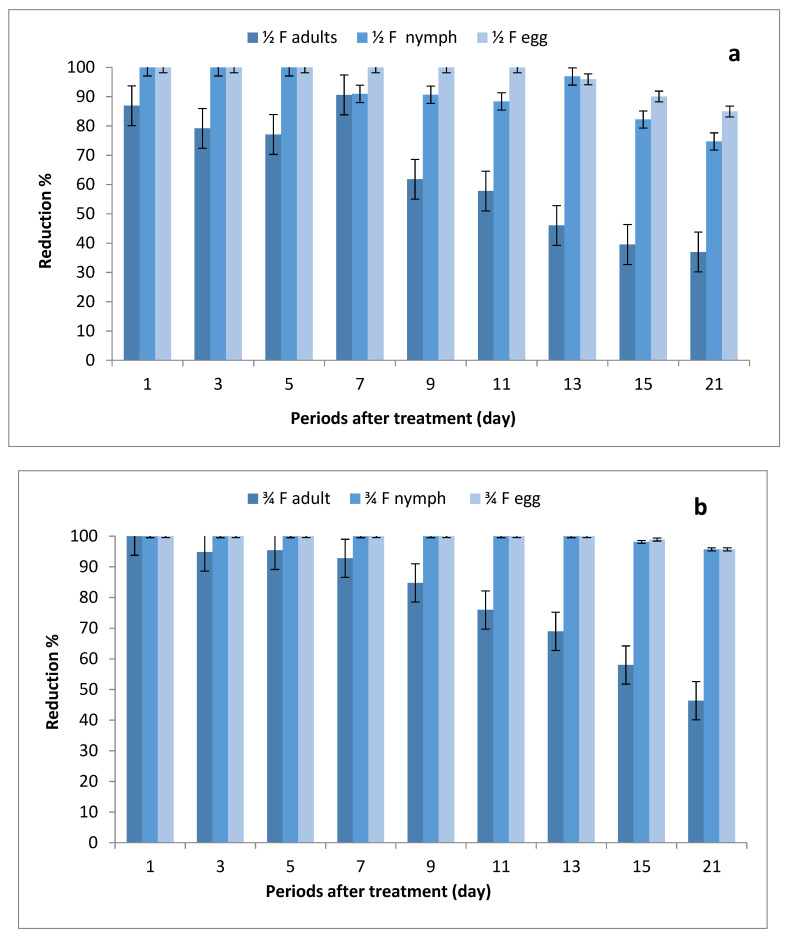

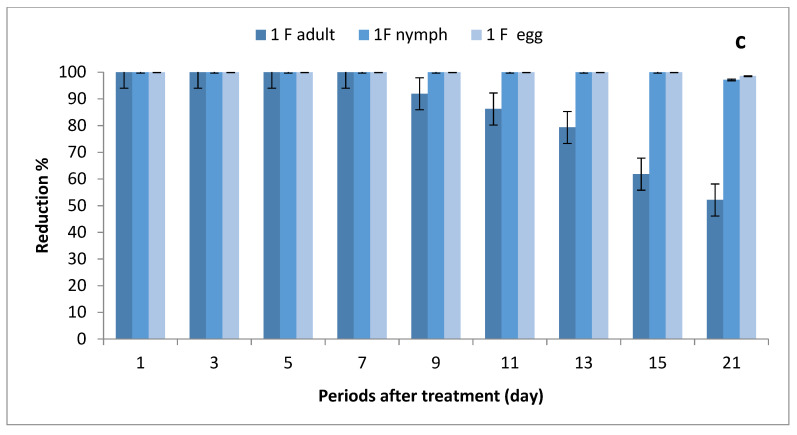
Effect of foliar spray of tomato STSs with 1/2 (**a**), 3/4 (**b**) and 1 (**c**) field rate of imidacloprid against *Bemisia tabaci* stages in field.

The data illustrated in Figure 2a–c indicate that there were significant differences in the number of eggs, nymphs and adults between STSs at all rates of imidacloprid and control, where the reduction percentages of whitefly eggs were (84.95–100%), (95.69–100%) and (98.53–100%) after being sprayed with ½, ¾ and 1 field rates, respectively. The reduction percentages in the number of nymphs were (74.10–100%), (95.67–100%) and (97.11–100%), respectively, while the reduction percentages in adult stages ranged between (36.97–86.90%), (46.35–100%) and (52.14–100%), respectively.

Effect of three rates of imidacloprid foliar sprays on tomato seedlings produced from untreated seeds against B. tabaci in the field:

In this experiment, the effect of tomato seedling produced from untreated seeds (SUSs) and sprayed with ½, ¾ and 1 recommended rates on whitefly stages at different periods were evaluated.

**Figure 3:**
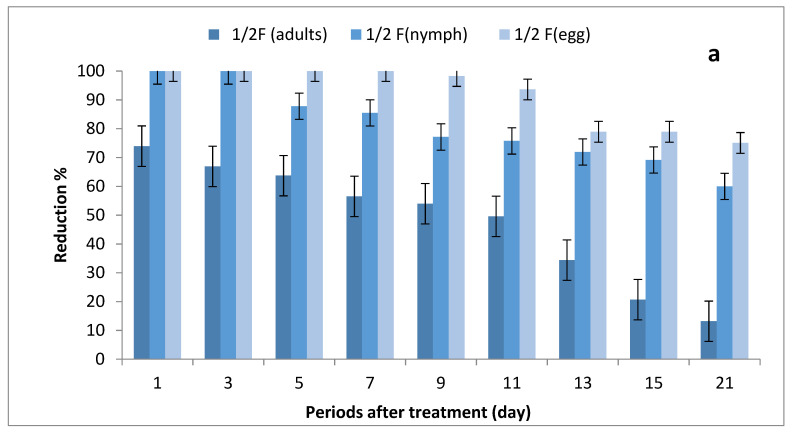

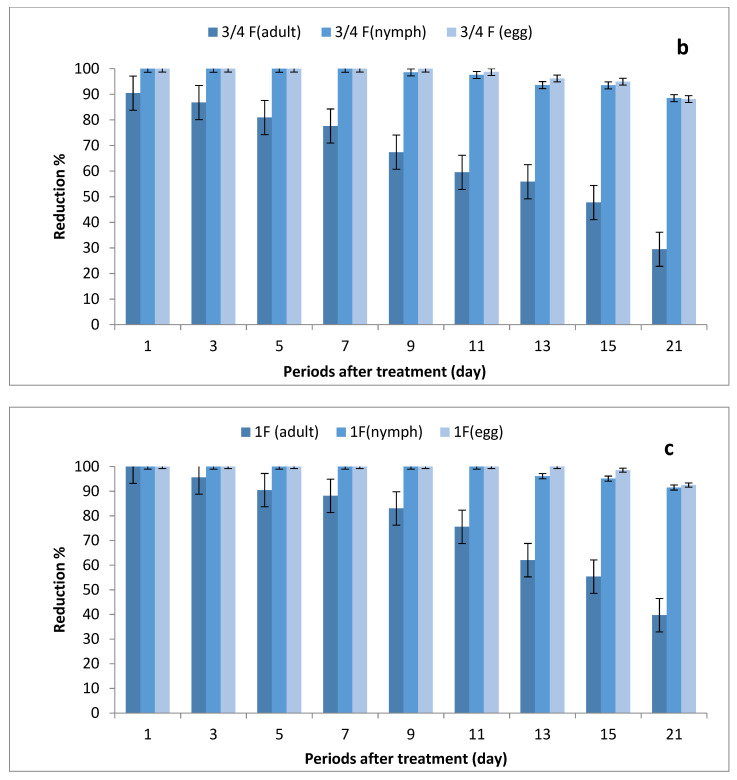
Effect of foliar spray of tomato seedlings produced from untreated seeds with 1/2 (**a**), 3/4 (**b**) and 1 (**c**) field rate of imidacloprid against *Bemisia tabaci* stages in field.

The results depicted in Figure 3a–c show the effect of ½, ¾ and 1 field rates of imidacloprid sprayed on SUSs against *Bemisia tabaci* stages on tomato plants. There were no significant differences in grand mean numbers of whitefly egg between ½ and ¾ rates and between ¾ and 1 field rate, while there were significant differences among the three rates in nymph numbers; furthermore, there were no significant differences between ½ and ¾ rates and between ¾ and 1 field rates in adult numbers, where there were significant differences between ½ and 1 field rate.

The reduction percentages in whitefly eggs were (75.08–100%), (88.16–100%) and (92.52–100%) for ½, ¾ and 1 field rates, respectively; (60–100%), (88.48–100%) and (91.52–100%) in nymph stages; and (13.18–73.96%), (29.46–90.45%) and (39.68–100%) for adult stages.

Generally, reduction percentages of whitefly stages in tomato STSs sprayed with ½, ¾ and one field rates of imidacloprid were more than the tomato SUSs sprayed with the three rates of imidacloprid; in addition, the reduction percentages in egg and nymph stages were more than those of adults in all periods at the three tested rates. Meanwhile, spraying with 1 field rate of imidacloprid increased the reduction percentages of egg, nymph and adult stages of whitefly compared with the ½ and ¾ field rates.

Compare with our previous work [17], which compared the effect of untreated seedlings and those sprayed with 1/2 and 1, to recommend the rate on whitefly stages.

The obtained results are in harmony with those of [28], which revealed that imidacloprid at 175 mL/ha achieved 100% control of the whitefly population on tomato at 5 days after spraying and that the population decreased to minimum numbers at 10 and 15 days after spraying. Additionally, [29] found that the foliar application of Actara and Confidor significantly decreased whitefly stages infecting eggplant at one day of treatment. In addition, [6] reported that neonicotinoid insecticides play an effective role in management of *B. tabaci* on vegetables. Furthermore, [30] stated that imidacloprid under field conditions induced high reduction in adults and immature stages of the sweet potato whitefly, *Bemisia tabaci*, which ranged from 83.19–93.24% and 77.02–82.48%, respectively. Moreover, [31] reported that imidacloprid used as seed treatment or a foliar spray in combination with other pesticides demonstrated a significant effect and caused great reduction in the whitefly population as follows: the combination of carbofuran (used as soil application) + imidacloprid (as seed treatment) + imidacloprid (as foliar application) was the highest effective combination for reduction of the whitefly population, followed by imidacloprid (as seed treatment) + thiamethoxam (spray), imidacloprid (as seed treatment) + imidacloprid (foliar spray), imidacloprid (as seed treatment) + dimethoate (foliar spray), carbofuran (as soil application) + malathion (foliar spray), and imidacloprid (as seed treatment) + yellow sticky traps. It was found by [32] that foliar spray of the recommended rate of imidacloprid on cotton under field conditions achieved a high reduction percentage on *B. tabaci* adults (73.39 and 75.43%) and immature stages (72.24 and 86.60%) in the 2012 and 2013 cotton seasons. In addition, [23] found that foliar spray of imidacloprid insecticide on cucumber plants greatly reduced the population of *B*. *tabaci* after 7 days of treatment.

### 2.1. Effect of Imidacloprid on Tomato Fruit Yield

In this experiment, the effect of tomato of seedling produced from treated roots (STRs)sprayed with ½, ¾ and 1 recommended rates on tomato fruits was compared with the effect on tomato seedling produced from untreated seeds (SURs) sprayed with the three rates in order to find out if the treatment of tomato seeds before planting had an effect on increasing the yield compared to seedlings whose seeds were not treated, as well as which of the three treatments achieved the highest yields.

Regarding our previous work, [17], which compared the effect of STRs with 1/2 and 1 recommended rates of imidacloprid and SURs sprayed with two rates of imidacloprid on tomato yields without any seed treatments: Statistical analysis of the obtained results in Table 1 revealed that there were no significant differences between STRs and SURs sprayed with ½ recommended rate (RD) of imidacloprid, and the same trend was noticed with ¾ RD and 1 RD, while there were significant differences between STRs and SURs sprayed with ½ RD and all other treatments (LSD 5% = 340.97). The RSD% ranged from 0.13 to 0.40% for STRs sprayed with three rates and from 0.28 to 0.35% for SURs that were sprayed. As for the increase in percentage in tomato yield compared with the control, it was obvious that the increase in percentage ranged from 31.38 to 56.71% and from 23.30 to 51.42% for STRs and SURs, respectively.

Generally, treating tomato seeds before planting had no significant effect on fruit yield compared with untreated seeds, while there were significant differences in this direction between ½ RD and the other two treatments. The amount of yield increased in STRs and SURs sprayed with imidacloprid, compared with the control.

The obtained results are in agreement with [33], who found that foliar spraying with imidacloprid on tomato plants under field conditions reduced the number of sprays and tomato yield increased when under field conditions. Additionally, [34] stated that imidacloprid increased the tomato fruit yield compared to the control when used against *Tuta absoluta*. In addition, [35] found that the highest yield and lowest content of mean pesticide residue (0.054%) was recorded in plots treated with imidacloprid.

### 2.2. Recovery of Imidacloprid

The results in Table 2 show the recovery percentages of imidacloprid in tomato leaves and fruits. It was 102.2–102.7 where the LOD and LOQ level were 0.010 and 0.030 mg/kg. The RSD% value ranged from 2.94 to 9.86% and from 1.17 to 9.89% for leaves and fruits, respectively.

The obtained results are in agreement with those conducted by [36], who found that the recovery percentage of imidacloprid ranged between 103.2 and 113% in tomato fruits. Additionally, [37] recorded that imidacloprid recovery spiked from mango, cowpea and water ranged between 90 and 110%.

*Imidacloprid residues in tomato leaves and fruits of seedlings produced from treated seeds and sprayed with one field recommended rate:*

This experiment was conducted to determine the residues of imidacloprid in tomato fruits and leaves of STSs sprayed with recommended rates compared with tomato fruits and leaves produced from untreated seeds at different periods of treatment to find out the residues and disappearance rate of imidacloprid in fruits and leaves, as well as the PHI and MRL of imidacloprid. (The residues of pesticide were determined in the leaves because the insect feeds on the sap of the leaves, and the numbers of the different stages of the insect were also counted periodically after spraying to know how to gain the greatest control with the least bad effects to humans and the environment.) As for our previous work, [17] compared the residues of imidacloprid in the leaves and fruits of SURs and seedlings produced from untreated roots at different periods, without any treatments on the seeds.

**Figure 4:**
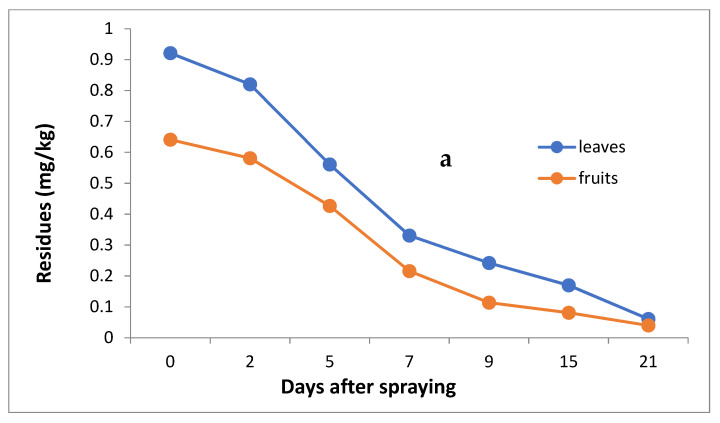

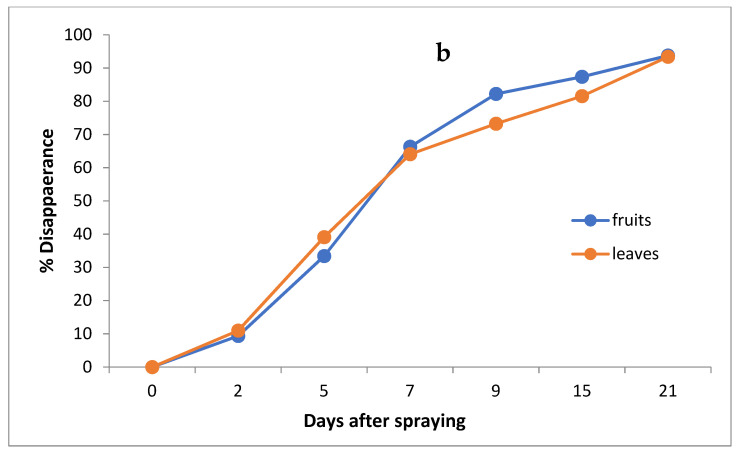
Residues (**a**) and % disappearance (**b**) of imidacloprid in the leaves and fruits of seedlings produced from treated seeds sprayed with field recommended rate.

The data in Table 3 and Figure 4a,b indicate that the initial residues one hour after imidacloprid application were 0.921 and 0.641 mg/kg in the leaves and fruits, decreasing to 0.820, 0.561, 0.331, 0.242, 0.170 and 0.061 mg/kg for leaves and 0.581, 0.427, 0.216, 0.114, 0.081 and 0.040 mg/kg for fruits after 2, 5, 7, 9, 15 and 21 days, respectively.

However, the results show that loss percentages after 2, 5, 7, 9, 15 and 21 days of treatment were 10.97, 39.09, 64.06, 73.24, 81.54 and 93.38% and 9.36, 33.39, 66.30, 82.22, 87.36 and 93.76% for leaves and fruits, respectively, with R^2^ = 0.82 and 0.86 for leaves and fruits, respectively. The half-life time (t ½) was 6.99 and 6.48 days for leaves and fruits. The RSD percentage value ranged from 1.19 to 7.13% for leaves and from 1.39 to 6.17% for fruits.

*Imidacloprid residues in tomato leaves and fruits in seedlings produced from untreated seeds and sprayed with one field recommended rate:*

**Figure 5:**
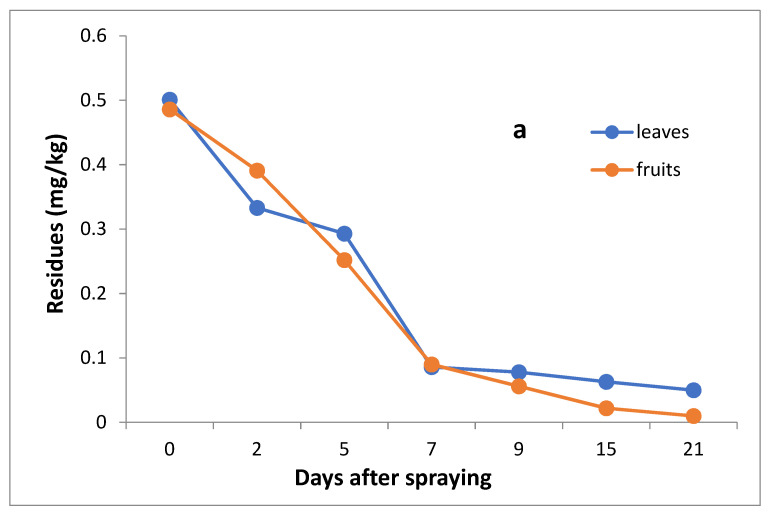

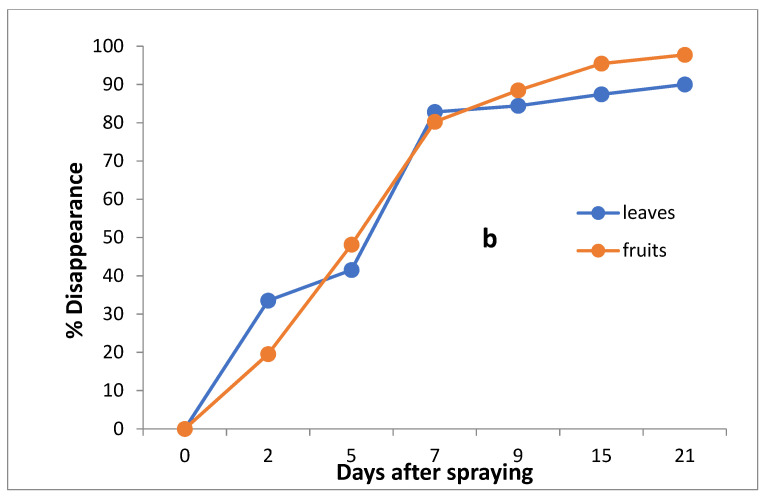
Residues (**a**) and % disappearance (**b**) of imidacloprid in the leaves and fruits of seedling produced from untreated seeds and sprayed with field recommended rate.

The data in Table 3 and Figure 5a,b indicate that the initial residues one hour after imidacloprid application in leaves and fruits were 0.501 and 0.486 mg/kg, decreasing to 0.333, 0.293, 0.086, 0.78, 0.063 and 0.050 mg/kg for leaves and 0.391, 0.252, 0.130, 0.056, 0.022 and 0.10 mg/kg for fruits after 2, 5, 7,9,15 and 21 days, respectively.

However, the results show that the loss percentages after 2, 5, 7, 9, 15 and 21 days of treatment were 33.53, 41.52, 82.83, 84.43, 87.43 and 90.02% for leaves and 19.55, 48.15, 73.25, 88.48, 95.47 and 97.74% for fruits, with R^2^ = 0.91 and 0.89 for leaves and fruits, respectively. The half-life time (t ½) was 5.59 and 4.59 days for leaves and fruits, respectively. The RSD% value ranged from 3.00 to 11.63% for leaves and from 1.59 to 10% for fruits.

It could be reported that the initial residues of imidacloprid in fruits of seedlings produced from treated seeds and others produced from untreated seeds sprayed with imidacloprid were less than the maximum residual level (MRL = 1 mg kg^−1^) recommended by the Codex Alimentarius Commission, as well as less than the American and Canadian tolerance level (MRL = 1 mg kg^−1^) [38].

The obtained results are in agreement with those of [21], who found that imidacloprid has a minimum preharvest interval (PHI) and was more toxic against sweet potato whitefly adults, so it can be recommended for effective and safe use in the control of the sweet potato whitefly on tomato. Additionally, [39,40,41] reported that the residues of imidacloprid were rapidly lost in grape leaves in 21 days of its application at the recommended dose. Furthermore, [42,43] found that the amount of imidacloprid quickly decreases in the first few hours/days after application because its residues are rapidly lost from plant surfaces via volatilization or in a few different manners. It was found by [20] that the residues of imidacloprid dissipated below the maximum residue limit (MRL) of 1 mg kg^−1^ in 3 days in cucumber fruits. In addition, [44] found that the degradation of imidacloprid was relatively higher on brinjal fruits. Completely consistent with our results, [45] found that imidacloprid amounts sharply decreased after the first day of application and reached 72.3% from the initial deposit on tomato fruits; the initial residue of imidacloprid in the tomato fruits was 0.650 ppm and the estimated preharvest interval for imidacloprid was 2 days after application on tomato plants. In addition, [46] found that the initial deposits of imidacloprid on tomato fruits were 0.643 mg/kg, while the half-life value of imidacloprid was 2.91 days. Additionally, [17] found that the residues of imidacloprid in tomato fruits produced from treated seedling roots and untreated plants sprayed with field rate were less than the MRL = 1 mg kg^−1^ recommended by the Codex Alimentarius Commission and the pre-harvest interval (PHI) was 1 day, with a half-life time (t ½) of 7.27 and 6.37 days in the tomato leaves and fruits in root-treated seedlings sprayed with the field recommended rate, respectively, and the half-life time was 7.14 and 4.88 days in tomato leaves and fruits of untreated root seedlings sprayed with the field recommended rate, respectively. Moreover, [47] reported that the residue levels of imidacloprid gradually decreased in grape berry samples collected after the insecticide application for 21 days. It was reported by [6] that neonicotinoid insecticides played an effective role in the management of *B. tabaci* on vegetables. Recently, [48] found that the average initial deposits (2 h of application) were 0.227 mg/kg at the recommended dose of imidacloprid in tomato fruits with 1 day as the pre-harvest interval. Contrary to our results, [49,50] found that the residues of imidacloprid in tomato samples collected from the greenhouse in the initial days were higher than their MRL.

## 3. Materials and Methods

### 3.1. Chemicals and Tested Insecticide

Imidacloprid (Admire 20% SC) (1-[(6-chloro-3-pyridyinyl) methyl]–N-nitro-2-imidazolidinimine)) was obtained from Bayer Company in Egypt. The standard of imidacloprid (99.9% purity) was provided from Sigma-Aldrich. All solvents were HPLC grade and purchased from pharmaceutical companies in Egypt.

### 3.2. Field Experiments and Sampling Procedure

Complete randomized design experiments with three replicates were conducted at a private farm of Elbehira Governorate, Egypt, during the period from March 2021 to October 2021.

Fifty plots of land were prepared (each 20 m^2^). Each plot was divided into four rows consisting of 10 m length and 0.5 m width. Tomato seeds, *Lycopersicon esculentum* Miller, Variety Super Strain B were planted in the nursery, where 20 g of seeds were used. The row was cultivated on both sides at a planting distance of 20 cm in the upper third and covered with silt or sand. Seedlings (30 days old) were planted at requested plots. After thirty days of transplanting, 35 g of sodium sulfate + 12 g of potassium sulfate + 52.5 g superphosphate/plot) were added. One month later, 52.5 g sodium sulfate + 24 g potassium sulfate/plot were added. Moreover, after 3 months, 35 g ammonia nitrate + 24 g potassium sulfate/plot were supplied. Finally, after the first fruit appearance, 35 g ammonia nitrate/plot were added.

### 3.3. Seed Treatment with Imidacloprid

Three plots were planted with seedling produced from treated seeds (STSs) soaked for 5 min in insecticide solution (3 mL/one liter water).

Two weeks after planting, twenty-five tomato leaves were randomly collected every week from each replicate early in the morning and transported to the laboratory for examination. The numbers of whitefly adults were counted in the field early in the morning before flight activity, while whitefly egg and nymph stages were counted in the laboratory using the dissecting microscope 12 weeks after planting. The reduction percentages were calculated using the Abbott formula [51]:

R % = (1 − no. in treated after treatment/no. in control after treatment) × 100

where, R = reduction, no = number of insect stages.

### 3.4. Foliar Spraying with Imidacloprid

Nine plots were cultivated with SUSs and nine plots were also cultivated with tomato STSs, then each three plots were sprayed two months after planting (only single spray) with imidacloprid 20% SC using a calibrated hand-held compression sprayer (Kwazar), at 1 field rate (125 mL/100 L water), ¾ field rate (93.75 mL/100 L water) and ½ recommended rate (62.5 mL/100 L water) while the other three plots were sprayed with water and left as control. To prevent contamination with foliar spray of imidacloprid and interference during the experiment processes for all experimental plots, two rows of land between treatments were left without plants as a barrier. Samples of 25 leaves per replicate were randomly collected before spraying and at 1, 3, 5, 7, 9, 11, 13, 15 and 21 days after pesticide spraying. The numbers of whitefly adults, eggs and nymphs were counted as previously mentioned. Reduction percentages of whitefly stages were determined according to the equation of [52].

R% = [1 − (n in C before T ∗ n in T after T/n in C after T ∗ n in T before T) ∗ 100]

where, R = reduction, C = control, T = treatment.

The experiments were in a completely randomized design using three replicates.

### 3.5. Residues and Loss Percentage of Imidacloprid in Tomato Leaves and Fruits

Three plots were cultivated with the seedlings produced from the treated seeds with imidacloprid 20% SC solution (3 mL/1 L water) and the other three plots were cultivated with seedlings produced from untreated seeds.

Plots were sprayed with imidacloprid at one field rate (125 mL/100 L water) at fruiting period (only single spray).

One kg of tomato fruits and 100 tomato leaves were randomly collected from each treatment at one hour (zero time), 2, 5, 7, 9, 15 and 21 days after spraying the insecticide, and transferred to the laboratory where they were subjected to analysis in order to determine the imidacloprid residues.

### 3.6. Analytical Processes

Extraction and clean up:

Fifty grams of tomato fruits and other from leaves were randomly taken, cut into small pieces and put in the electrical blender. Then, 200 mL of acetonitrile were added. After blending for 5 min at high speed, the mixture was vacuum-filtered through a 12 cm Buchner filter. The filtrate was conveyed into a 500 mL separating funnel and then 10 mL of phosphate buffer solution (pH 7) was added; the separating funnel was then shaken strongly for 1 min, after which the acetonitrile phase was passed through a layer of anhydrous sodium sulfate on glass-wool and evaporated via a rotary at 40 ºC in a water bath. The dried extract was dissolved in 5 mL of acetonitrile: water (1:3), then sonicated for 5 min and filtered using a 0.2 µm filter for HPLC analysis [53].

### 3.7. Measurement and Residues via HPLC

The estimation of imidacloprid residues was determined on a Perkin Elmer (series 200) HPLC coupled with a diode array UV detector at 270 nm. Approximately 20 µL of sample were injected into a Nucleosil 100-5 reverse phase (C18) 5 µm, 250 × 4 mm column. Under the mobile phase of acetonitrile: water (25/75 *v*/*v*), the retention time of imidacloprid was 4.8 min.

Standard curve

**Figure 6:**
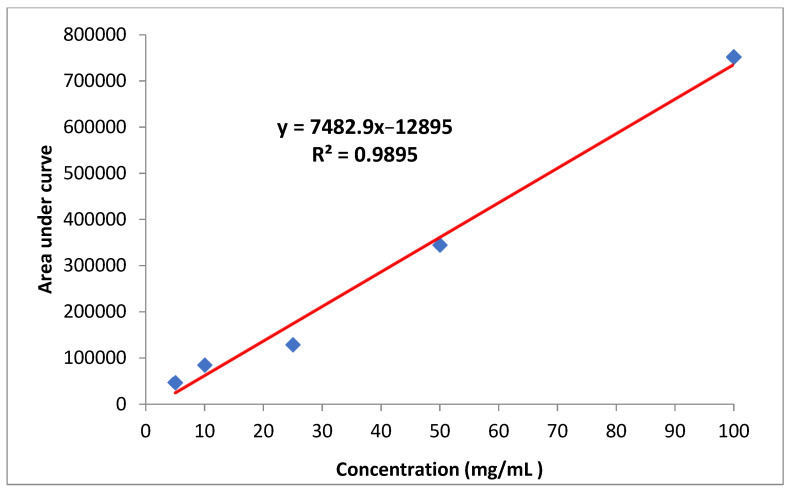
Standard curve of imidacloprid.

The stock solution of imidacloprid (1000 mg/L) was dissolved in acetonitrile and diluted to produce working standard solutions of 5, 10, 25, 50 and 100 mg/L. Calibration curves were generated by plotting peak area versus its concentration (Figure 6). All solutions were stored in a refrigerator at 4 °C.

### 3.8. Validation Procedure

The recovery experiments were carried out by spiking tomato at 0.25, 0.5 and 1 mg/kg where it was processed as previously described and analyzed via HPLC. This was replicated three times for each fortified concentration to establish the reliability and validity of the analytical method adopted. The calibration curve and tomato fortified samples were prepared using standard solutions. The limit of detection (LOD mg/kg) and limit of quantification (LOQ mg/kg) were evaluated on the basis of 3:1 (LOD) while LOD was determined on the basis of a signal-to-noise ratio of 10:1 [54,55].

### 3.9. Half-LIFE Time Value

Half-life time (t_1/2_) in days was calculated according to [56]

t _1/2_ = ln _2_/k = 0.693/k, k (apparent rate constant) = 1/t × ln a/m

where t = time in days, m = residue at x time and a = initial residue.

### 3.10. Effect of Imidacloprid on Tomato Yield

To conduct this experiment, nineteen experimental plots were prepared. Nine plots were cultivated with SUSs, where three of these plots were sprayed with imidacloprid at ½ the recommended field rate, another three plots were sprayed with ¾ the recommended rate and the last three plots were sprayed with one recommended rate using a calibrated hand-held compression sprayer (Kwazar). In addition, the other nine plots were cultivated with STSs, where three plots were sprayed with the ½ rate, three sprayed with the ¾ rate, and the other three plots were sprayed with one recommended rate, while the last three plots were sprayed with water and served as the control. Randomly, tomato fruits at a rate of 10 plants/treatment were weighed to determine the average weight of fruits/plant (g) and percent increase of yield.

### 3.11. Statistical Analysis

The obtained data were statistically analyzed using analysis of variance (ANOVA) at 5% probability, and the measurements were divided using Duncan’s multiple range test via a CoStat software program (Version 6.400) 1989–2008 [57]. The efficacy was computed using [51,52].

## 4. Conclusions

The treatment of tomato seeds with imidacloprid achieved high efficacy in controlling whitefly stages. The seedling produced from treated seeds (STS) and sprayed with three imidacloprid rates were more effective on whitefly stages than seedling produced from untreated seeds (SUSs) andsprayed with three rates; moreover, the STSs sprayed with one recommended rate was more effective against whitefly stages than that sprayed with ½ and ¾ the recommended rate. The STSs and sprayed with one recommended rate of imidacloprid achieved the highest tomato fruit yields compared with other treatments and the control. The residue in tomato fruits were less than the MRL, and the pre-harvest interval (PHI) was 1 day. In SUSs, the correlation coefficient R^2^ values were 0.91 and 0.89 for leaves and fruits, respectively, and the half-life time (t ½) was 5.59 and 4.59 days for leaves and fruits, respectively. In STSs, the R^2^ values were 0.82 and 0.86 for leaves and fruits, respectively, and the half-life time (t ½) was 6.99 and 6.48 days for leaves and fruits, respectively. The residues in leaves were more than in fruits.

Imidacloprid is effective for controlling whiteflies and its pre-harvest period is 1 day, therefore it is safe for consumers and can be recommended for use in integrated whitefly management programs.

## Figures and Tables

**Table 1:** Effect of spraying three rates of imidacloprid on seedlings produced from treated tomato seeds and seedlings produced from untreated seeds on fruit yield under field conditions.

Field Rates	Seedling Produced from Treated Seeds + Spraying (STSs)			Seedling Produced from Untreated Seeds + Spraying (SUSs)		
	Average Weight Fruits/Plant (g)	* Increase %	RSD %	Average Weight Fruits/Plant (g)	* Increase %	RSD %
½ RD treatment 1	921 bc ± 1.15	31.38	0.13	824 c ± 2.89	23.30	0.35
¾ RD treatment 2	1380 a ± 5.20	54.20	0.38	1240 ab ± 3.46	49.03	0.28
1 RD treatment 3	1460 a ± 1.15	56.71	0.40	1301 a ± 4.04	51.42	0.31
LSD 5%	340.97					

The different letters mean significant difference at the 5% level; RD: recommended rate; *: percentage increase = treated − control/treated × 100; RSD%: relative standard division.

**Table 2:** Recovery percentages of imidacloprid from tomato leaves and fruits.

Applied Amount (mg/kg)	Found Amount (mg)				Recovery %		Recovery Average %	
	Leaves ± SE	RSD%	Fruits ± SE	RSD%	Leaves	Fruits	Leaves	Fruits
0.25	0.259 c ± 0.013	8.49	0. 0.261 c ± 0.006	3.83	103.6	104.4	102.2	102.7
0.50	0.510 b ± 0.009	2.94	0.513 b ± 0.003	1.17	102.0	102.6		
1.00	1.014 a ± 0.058	9.86	1.011 a ± 0.058	9.89	101.0	101.1		
LSD 5%	0.018		0.013		-	-	-	-

Values followed by different letters are significantly different at the 5% level.

**Table 3:** Imidacloprid residues and disappearance percentage in the leaves and fruits of tomato produced from treated and untreated seeds with one field recommended rate.

Days After Spraying	Seedlings Produced From Treated Seeds + Spraying (STSs)			Seedlings Produced From Untreated Seeds + Spraying (SUSs)		
	Conc. ± SE (mg/kg)	RSD %	Disappearance %	Conc. ± SE (mg/kg)	RSD %	Disappearance %
Leaves						
1 h	0.921 a ± 0.006	1.19	-	0.501 a ± 0.021	7.19	-
2 days	0.820 b ± 0.029	6.10	10.97	0.333 b ± 0.006	3.00	33.53
5 days	0.561 c ± 0.023	7.13	39.09	0.293 c ± 0.006	3.41	41.52
7 days	0.331 d ± 0.006	3.02	64.06	0.086 d ±0.006	11.63	82.83
9 days	0.242 e ± 0.006	4.55	73.24	0.078 e ± 0.003	6.41	84.43
15 days	0.170 f ± 0.006	5.88	81.54	0.063 f ± 0.002	4.76	87.43
21 days	0.061 g ± 0.0006	1.64	93.38	0.050 g ± 0.001	4	90.02
LSD 5%	0.0096			0.0075		
t ½ (day)	6.99			5.59		
Fruits						
1 h	0.641 a ± 0.012	3.28	-	0.486 a ± 0.015	5.49	-
2 days	0.581 b ± 0.12	3.44	9.36	0.391 b ± 0.006	2.56	19.55
5 days	0.427 c ± 0.006	2.34	33.39	0.252 c ± 0.002	1.59	48.15
7 days	0.216 d ± 0.002	1.39	66.30	0.130 d ± 0.006	7.69	73.25
9 days	0.114 e ± 0.001	1.75	82.22	0.056 e ± 0.003	8.93	88.48
15 days	0.081 f ± 0.003	6.17	87.36	0.022 f ± 0.001	9.09	95.47
21 days	0.040 g ± 0.0006	2.75	93.76	0.010 g ± 0.0006	10	97.74
LSD 5%	0.008			0.0075		
t ½ (day)	6.48			4.59		
PHI (days)	1			1		

Values followed by different letters are the significant difference at 5%. Level PHI: pre-harvest intervals.

## Data Availability

The datasets used and/or analyzed during the current study are available from the corresponding author on reasonable request.

## Sample Availability

Samples of the compounds are available from the authors.

## Abbreviations

PHI: pre harvest intervals. MRL: maximum residual limits. t ½: half-life time. STR: seedling produced from treated roots. SUR: seedling produced from untreated roots. STS: seedling produced from treated seeds. SUS: seedling produced from untreated seeds RD:recommended rate.
